# Proceedings Twelfth Annual Meeting Am. S. Dental Surgeons

**Published:** 1852-01

**Authors:** 


### THE PROCEEDINGS

*Of the Twelfth Annual Meeting of the American Society of Dental Surgeons, held in Philadelphia on the 5th, 6th, and 7th of August,* 1851.

[Reported for the Dental News Letter, by Prof. E. WEBSTER, Stenogropher.]

TUESDAY MORNING, August 5, 1851.

The association convened at Sansom Street Hall, at nine o'clock, and was called to order by the President, Dr. E. Parmly.

On motion of Dr. E. Townsend, the dentists of Philadelphia were invited to attend the sittings of the society.

Reports from the several officers were read, and other private business transacted.

Committee on Aphorisms reported their readiness to bring the result of their labors before the association.

A committee was appointed to take into consideration the propriety of altering the constitution, with power to alter or amend the same, and report. Committee—Drs. Townsend, Foster, and Arthur.

A committee was appointed to confer with the Treasurer, and report such names of members who had forfeited their membership, agreeable to the constitution, by the non-payment of annual dues.

At the request of a member, his name was stricken from the roll.

The Secretary was instructed to prepare a notice, for publication in the newspapers, inviting the public to be present at the meeting on Wednesday, at 4½ o'clock, P. M., to hear addresses from members of the association.

Dr. Dunning then read his aphorisms on the treatment of exposed dental nerves. Adjourned till four o'clock, P. M.

---

AFTERNOON SESSION.

On motion, Dr. A. C. Hawes read his aphorism on the preservation of the teeth. Dr. A. Hill read a paper on obtaining plates by the elctro-type process. Dr. John Allen gave the history of his experience in the same field of experiment; also, on the subject of uniting single teeth to metallic plates by fusing a mineral attachment; specimens of which were exhibited, for which the society returned their thanks.

Committee on Membership reported, advising that all members in arrears for three years, and who, after notification, still neglect to make payment, be stricken from the roll; which was approved by the meeting.

After some business of a strictly private nature, in reference to membership and character, on which a committee was appointed, the association adjourned till Wednesday morning.

---

WEDNESDAY, 9 O'CLOCK, A. M.

Committee on character reported, which was laid on the table.

Dr. A. Hill, by appointment, then read an essay on artistic dentistry, for which the thanks of the society were returned him, and a copy asked for publication.

Dr. A. C. Hawes followed by an essay on Professional Empiricism, for which the thanks of the association was tendered him, and a copy asked for publication.

President.—The remaining portion of our time will be occupied with discussions, and the first thing before us is a paper read by Dr. Dunning yesterday.*

Dr. Dunning.—I will state for the information of gentlemen not present yesterday that there are a few short remarks intended to give the views of the society when they have been digested by it, upon the subject of treating *exposed nerves.*

Dr. Arthur.—In regard to the first *Aphorism* there is known to be a difference of opinion in the profession, and I think it would be satisfactory to endeavor to ascertain what is the state of opinion in the society with regard to it. I propose, therefore, that members state their opinions on this subject.

Dr. Westcott.—I would like to ask whether one class of cases which are not mentioned here, was neglected intentionally or through oversight. I refer to cases where the nerve is in a state of absolute decomposition, the tooth containing only a semifluid substance.

Dr. Dunning.—I merely speak of cases where the nerve is exposed, and where the destruction of the nerve is a part of the operation.

---

*The Aphorisms offered by Dr. Dunning, were not furnished to the reporter but the substance of the two first, and most important, are as follows: "A tooth whose nerve is exposed, may be permanently saved by entirely removing the pulp and filling the place occupied by it with gold."

Dr. Arthur.—I would state that for a number of years I have been satisfied of the utility of this operation, and have practised it frequently without hesitation. When a person presents himself with the pulp of a tooth exposed and in a healthy condition, I am so confident of success that I hardly think it necessary to state to him that there is more than a remote possibility of failure.

Dr. White.—Is it meant by "permanently saved" that the tooth will be retained in the mouth as well as any other tooth?

Dr. Arthur.—I am not prepared to say. By permanently saved I mean that the tooth so treated will remain in a healthy condition for a great many years, an indefinite time.

Dr. Dunning.—None of us would be able to testify to the satisfaction of this meeting on that subject, but my feeling when I am called upon to treat such cases is entirely similar to that of Dr. Arthur's. I have treated such cases for ten years, and I was lead to treat them in that way by Dr. Maynard of Washington, and he, so far as I am concerned, was the originator of it. For many of the first years of my practice it was always a matter of doubt and uncertainty, and I did it with a great deal of hesitation, which I always took great care to express to the patient, that he might be made to go halves with me in the risk, and might feel, if there should be a failure, that he had not been deceived. But for some four, five or six years past I have lost a great deal of the feeling on the subject, and when the nerve is exposed, I have the same certainty as to the degree of success that I can attain in a given case, as I have in any ordinary operation; as, for instance, plugging a tooth; and I can say, and those who have been acquainted with my practice can bear me out, that I have failed in as few cases in this operation as the profession generally fail in any operation connected with the art. With regard to the permanency of it, I have not as yet fully tested it, various accidents have befallen such teeth; sometimes there will be, in the course of four or five years, the formation of an abscess which may have arisen from accidental causes, but there are many cases which I can call to mind, in which teeth have been treated in this way, and have continued four, five and six years without any abscess or ulceration, and where no discharge has taken place whatever.

Dr. White.—I only want to push my inquiries a little further. It seems to me the first proposition is the most important one, for it settles the point that it is proper and right to destroy the nerve. I am as willing to destroy the nerve as any one, yet this Aphorism would seem to imply, that that was the *only* method of saving the tooth. I have seen teeth treated as well where the nerve was not destroyed, as where it was; I have seen a case where a tooth was plugged twenty-five years over the nerve. I would like to have it more clearly established what is meant by this Aphorism, whether it means that the destruction of the nerve is the only treatment; or is it probable that we can frequently succeed without it.

Dr. J. Allen.—With reference to capping nerves, my experience does not tally with that of Dr. White, to the extent that he has mentioned. I have not been very successful in that course of treatment. I usually destroy the lining membrane of the tooth, by a fine, small, elastic instrument, so that I can cut it out at once. I prefer removing as large a portion of the lining membrane as possible. If there is only one fang, I can, generally, treat it successfully; if there are two, I have found more uncertainty in the operation; it is more difficult to cut out the lining membrane. If I find a tooth where the nerve is just exposed, I can treat it with success and confidence. The question arises, whether we can regard that as a permanent operation. I think in many cases we shall find that, after a few years, (frequently it will stand ten or fourteen years,) then an abscess will be formed, the fang is diseased and loosened. Can we regard that as permanent? So with regard to a plug put in a tooth, can we regard it as a permanent preservation of the tooth? I am under the impression that we cannot, though, in a majority of cases, where the nerve is just exposed, I believe such a tooth can be rendered useful for a number of years.

President.—I would remark, in reference to this Aphorism, which reads thus: "A tooth, the nerve of which is exposed, may be permanently saved,"—that the question now comes up, what does this mean—whether it is five, ten, or fifty years? A tooth may be successfully saved we know, and for many years; for I have seen many instances in which the success has been most complete, but whether it will last as long as life will last, the experience of none of us can establish. It is not many years since, in the practice of dental surgery, that it was thought, that when the nerve was exposed that the tooth was lost; but now, in our improved state of knowledge, we find that when a nerve is exposed, a tooth may be successfully, and, as far as our experience goes, can be permanently saved. The points we have now to consider are: what has been the experience in relation to the success of those who have practised it for the longest period of time, and what has been the success in the greatest number of operations.

Dr. Foster.—As far as my experience is concerned, I can only say, in regard to the exposure of nerves, that it has been my custom, where inflammation has been produced, never to attempt it under those circumstances. But where the nerve is exposed, or nearly so, but not sufficient to produce pain in the tooth, I have employed a cap to great advantage in a majority of cases. In cases where I have employed the probe, or other small cutting instrument, for the entire removal of the pulp, I have generally been successful; but where I have employed arsenic or cobalt, I have always felt extreme doubt as to the successful termination of that operation; but where I could use the instrument, I do not think a single case has come under my observation where suppuration has ensued, though there has sometimes been a slight inflammation. One of my instructors, Dr. Harwood, of Boston, not having read what was published on the excision of the nerve, previous to his return to the profession, made a publication, or a publication was made, of a discovery supposed to have been made by him on that subject, through which it has been his fortune, whether good or bad I will not say, to perform that operation probably more than any other man in the community for the last two years. I think he told me more than a hundred times in the latter part of the last year, and this year, I think, about seventy times. That is pretty extensive practice for that kind of operation. He said he had been successful, I think, with but two or three exceptions. Inflammation had not ensued, suppuration had not ensued, and he was still continuing to receive a great many of these cases. As far as my experience goes, in respect to the preservation of the teeth, under these circumstances, I can only say, that I believe that where that operation is thoroughly and well performed, the tooth is as likely to last as any living tooth, filled under ordinary circumstances. I think the chance of failure is less than with many living teeth, where the operator is obliged to take so much pains in the operation; and in all cases I would recommend excision in preference to the other.

[To be continued.]

